# The Invasive Caucasian Populations of the Brown Marmorated Stink Bug *Halyomorpha halys* (Hemiptera: Heteroptera: Pentatomidae) Rapidly Adapt Their Ecophysiological Traits to the Local Environmental Conditions

**DOI:** 10.3390/insects14050424

**Published:** 2023-04-29

**Authors:** Sergey Ya. Reznik, Margarita Yu. Dolgovskaya, Natalia N. Karpun, Vilena Ye. Zakharchenko, Aida Kh. Saulich, Dmitrii L. Musolin

**Affiliations:** 1 Zoological Institute of the Russian Academy of Sciences, Universitetskaya Nab. 1, 199034 Saint Petersburg, Russia; reznik1952@mail.ru (S.Y.R.); bcongroup@gmail.com (M.Y.D.); 2 Federal Research Centre the Subtropical Scientific Centre of the Russian Academy of Sciences, Yana Fabritsiusa Str. 2/28, 354002 Sochi, Russia; nkolem@mail.ru (N.N.K.); vilena.p2016@mail.ru (V.Y.Z.); 3 Department of Forest Protection, Wood Science and Game Management, St. Petersburg State Forest Technical University, Institutskiy Per. 5, 194021 Saint Petersburg, Russia; 4 Department of Entomology, Saint Petersburg State University, Universitetskaya Nab. 7–9, 199034 Saint Petersburg, Russia; 325mik40@gmail.com; 5 European and Mediterranean Plant Protection Organization, 21 Boulevard Richard Lenoir, 75011 Paris, France

**Keywords:** brown marmorated stink bug, diapause, geographic variation, *Halyomorpha halys*, invasive pests, local adaptations, microevolution, photoperiodic response, pre-adult development, reproduction

## Abstract

**Simple Summary:**

The ability to rapidly adapt to new environmental conditions is very important for the wide-scale invasion of pests or intentional introduction of beneficial insects. Diapause (i.e., a seasonal dormant state) formed in autumn in response to short days is a vital adaptation ensuring that the active development and reproduction of an insect population coincide with the favorable warm periods of the year. We conducted a laboratory study aimed to compare the induction of diapause in two invasive Caucasian populations of the brown marmorated stink bug *Halyomorpha halys,* which recently invaded two neighboring regions with subtropical (Sukhum, Abkhazia) and temperate (Abinsk, Russia) climates. Under the constant temperature of 25 °C and day lengths of 15.0 h and 15.5 h, the population from Abinsk showed a slower nymphal development and a stronger tendency to enter diapause than those from Sukhum. This finding agreed with the difference between the local dynamics of the autumnal temperature decrease. Similar interpopulation differences are known in other insects but our finding is distinguished by a very short adaptation time: the brown marmorated stink bug was first recorded in Sukhum in 2015 and in Abinsk in 2018. Thus, the differences between the compared populations evolved very quickly—during only several years.

**Abstract:**

The ability to rapidly adapt to new environmental conditions is a crucial prerequisite for the wide-scale invasion of pests or intentional introduction of beneficial insects. A photoperiodically induced facultative winter diapause is an important adaptation ensuring synchronization of insect development and reproduction with the local seasonal dynamics of environmental factors. We conducted a laboratory study aimed to compare photoperiodic responses of two invasive Caucasian populations of the brown marmorated stink bug *Halyomorpha halys* (Hemiptera: Heteroptera: Pentatomidae), which recently invaded neighboring regions with subtropical (Sukhum, Abkhazia) and temperate (Abinsk, Russia) climates. Under the temperature of 25 °C and the near-critical photoperiods of L:D = 15:9 h and 15.5:8.5 h, the population from Abinsk showed a slower pre-adult development and a stronger tendency to enter winter adult (reproductive) diapause compared to the population from Sukhum. This finding agreed with the difference between the local dynamics of the autumnal temperature decrease. Similar adaptive interpopulation differences in the patterns of diapause-inducing responses are known in other insect species but our finding is distinguished by a very short adaptation time: *H. halys* was first recorded in Sukhum in 2015 and in Abinsk in 2018. Thus, the differences between the compared populations might have evolved over a relatively short span of several years.

## 1. Introduction

Intraspecific variation observed both between and within natural populations affects all morphological, physiological, and behavioral traits of insects. These variations serve as a basis for adaptation to temporal changes and spatial heterogeneity of the environment. Moreover, intraspecific variability is the prerequisite to natural evolution and artificial selection of living beings. Most insects studied in this respect showed, although to a various degree, intraspecific variation in their responses to diapause-inducing environmental cues. Facultative diapause is one of the most important ecophysiological adaptations of many insects, ensuring synchronization of development, reproduction, and other activities with the seasonal dynamics of environmental factors. In contrast to quiescence that is directly induced and maintained by low temperature or some other unfavorable factors, facultative diapause can be considered as an ‘anticipatory response’. Facultative diapause is induced before the onset of a period of adverse conditions by environmental cues: photoperiod (day length), temperature, precipitation, air humidity, starvation, low quality food, etc. [1,2,3,4,5,6,7,8]. Therefore, photoperiodic, thermal, trophic, and other responses that induce facultative diapause and ensure survival in adverse seasons are of very high adaptive value. That is why all diapause-inducing responses are permanently controlled and (if necessary) changed by natural selection. In widely distributed insect species, this selection operates differently in different populations inhabiting regions with different climates. This resulted in the emergence of intraspecific (inter-population) variations which provide the basis for risk-spreading strategies, adaptations to the peculiarities of the local climate, and, finally, to micro- and macroevolution of seasonal adaptations [9,10,11,12].

Intraspecific variability in the induction of diapause is especially important for rapidly spreading invasive species which constantly face the necessity of adaptation to new environmental conditions. Patterns of seasonal dynamics of main abiotic environmental factors (i.e., temperature, precipitation, etc.) can differ markedly in the native and invasive ranges of an invader. Moreover, climate parameters can differ considerably among invaded regions. Therefore, an ability to quickly adapt to new environmental conditions is a crucial prerequisite for a successful wide-scale invasion or intentional introduction [13,14,15,16,17,18]. Such adaptive changes in photoperiodic and/or thermal diapause-inducing responses have been shown in several insect invaders and introduced biocontrol agents [19,20,21,22,23,24,25,26]. These earlier studies indicate that despite certain general regularities, adaptation patterns of individual insect species can differ markedly. Therefore, prediction of potential range and elaboration of control measures for each invasive insect require special studies.

The model species of our study, the brown marmorated stink bug, *Halyomorpha halys* (Stål, 1855) (Hemiptera: Heteroptera: Pentatomidae), is a polyphagous insect pest that originated in Eastern Asia (China, Korea, Japan, Myanmar, and Vietnam) and recently became one of the most harmful invasive insects in North America and Europe. This highly polyphagous pest causes severe damage to various agricultural and ornamental plants, resulting in substantial economic losses. Additionally, adult bugs seeking winter shelter often climb into houses, causing discomfort to humans [27,28,29,30,31,32,33]. It is known that in its both native and invasive ranges *H. halys* overwinters in the state of adult (reproductive) diapause induced by the long-day type photoperiodic response [29,32,33,34,35,36,37,38,39,40,41]. It was shown that the critical photoperiod (that is the day length, which induces diapause in 50% of the population) of individuals from the native Korean population significantly differs from that of the bugs from different invasive European populations [33].

In 2014, the brown marmorated stink bug was recorded on the coast of the Black Sea, in Sochi City [42], and, currently, the species has become rather widely spread from Abkhazia to the northern macro-slope of the Main Caucasus Range in Russia [32,33,43]. Most likely the invasion of *H. halys* into the region followed the invasion of the pest and its massive spread in Western and Central Europe during the preceding decade (see chronology of the invasion in [33]). It should be noted that although this invaded zone is so far comparatively small (ca. 400 km in length), the local climate gradually changes from wet subtropical in the south-eastern part (Sukhum, Abkhazia) to dry temperate in the north-western part (Abinsk, Krasnodar Krai, Russia) of the discussed region. Thus, the question arose: was this relatively small-scale spread of the brown marmorated stink bug along the north-eastern coast of the Black Sea accompanied by corresponding changes in the pattern of the photoperiodic induction of winter adult diapause, similar to what evolved during the large-scale invasion from Eastern Asia to Europe [33]? The present study was designed to address this question.

## 2. Materials and Methods

### 2.1. Insects, Collection Sites, and General Methods

**Figure 1:**
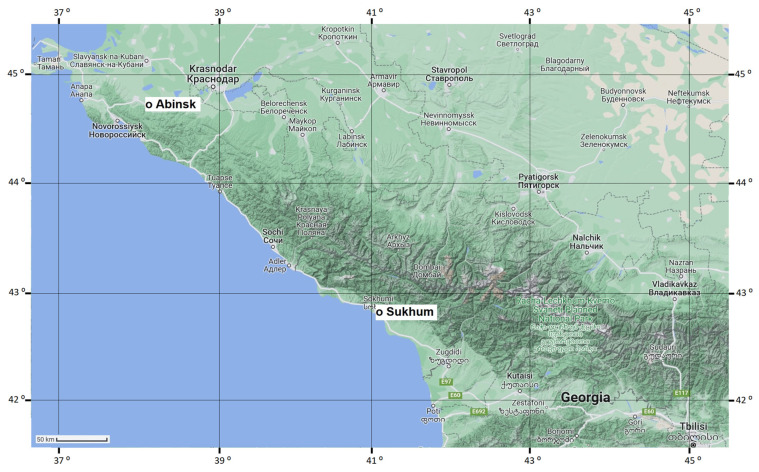
Locations of the collection sites: Abinsk and Sukhum. The map is based on an image from ©Google, 2023.

The study was conducted with two laboratory populations established from *H. halys* collected in September 2020 in the two following locations (Figure 1):

(1) Environs of Sukhum city, Abkhazia, ca. 42.95° N, 41.08° E, 20 m a.s.l., hereafter referred to as the Sukhum population;
(2) Abinsk, Krasnodar Krai, Russia, ca. 44.87° N, 38.16° E, 41 m a.s.l., hereafter referred to as the Abinsk population.

**Figure 2:**
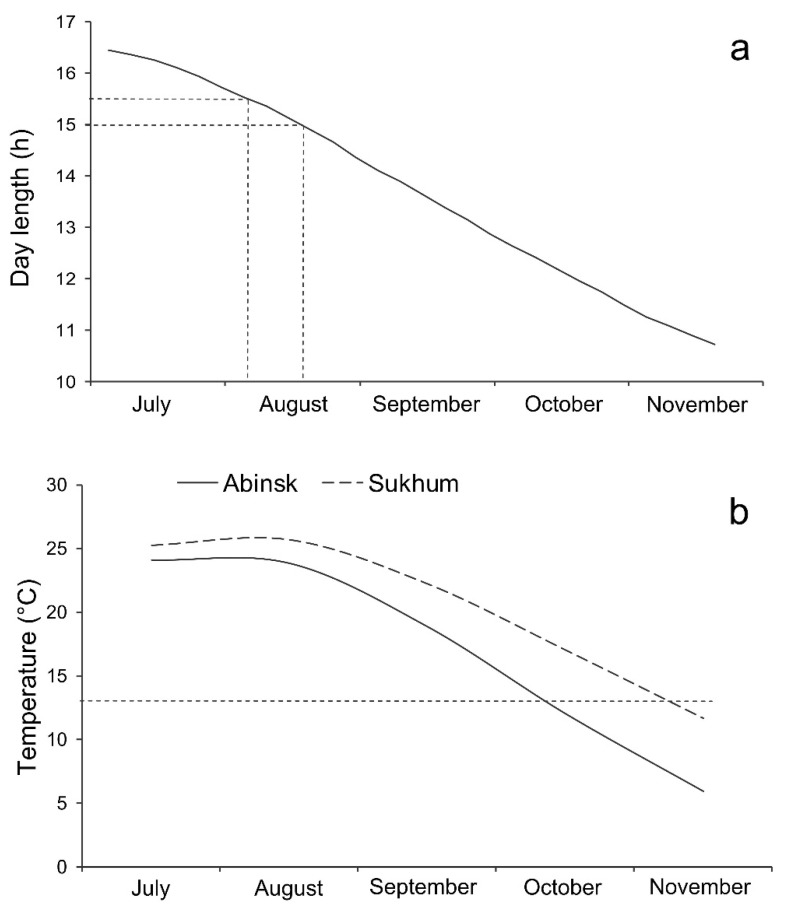
Seasonal dynamics of day length and temperature in Sukhum and Abinsk. (**a**) day length in Sukhum including civil twilight [47]; the dashed lines show the approximated critical day length of the Sochi population of *Halyomorpha halys* (15.0–15.5 h [33]); (**b**) mean monthly temperatures in 2011–2020 in Abinsk (data from the nearest weather station in Krymsk (ca. 44.93° N, 38.00° E, 21 m a.s.l. [48]) and Sukhum (data from the Hydro-Meteorological Service of the Republic of Abkhazia); the dashed line shows the lower development threshold of *H. halys* (13.3 °C) [39].

The annual dynamics of day length in Sukhum and Abinsk are similar (a difference never exceeds 15–20 min on the same dates) and, to avoid overlap, only the data for Sukhum are given in Figure 2a. The timing of the critical day lengths for diapause induction was estimated based on the data for the earlier studied close population from Sochi [32,33]. They fall in the first half or the middle of August.

Despite a rather short distance between the two collection sites (ca. 300 km; Figure 1), their local climates are quite different. The climate of Sukhum is wet subtropical (with a lingering spring, hot summer, and long warm autumn) [44,45]. Abinsk is in the steppe zone of temperate climate (with a short spring, hot dry summer, short autumn) [46]. The difference in the mean temperatures at the end of summer and the beginning of autumn is quite substantial, constituting ca. 1.9 and 3.4 °C in August and September, correspondingly (Figure 2b).

In Sukhum, *H. halys* was first recorded in 2015 [32,43]. Shortly thereafter, in 2018, the pest arrived at the Abinsk region, which is located on the northern macro-slope of the Main Caucasus Range in Russia [49].

For the laboratory experiments, about 100 adults were collected from each location to establish the laboratory populations. The experiments were conducted with the second laboratory generation resulting from the field collections. As in the previous study [33], before the experiments, insects in the laboratory populations were reared in ventilated transparent plastic containers (28 × 19 × 14 cm) at a temperature of 25–28 °C and diapause-averting photoperiod of L:D = 16:8 h (hereafter, L:D refers to the durations of photophase (light period) and scotophase (dark period) in hours). *Halyomorpha halys* nymphs and adults fed on broad bean (*Vicia faba* L.) seedlings, peanuts (*Arachis hypogaea* L.), sunflower (*Helianthus annuus* L.) seeds, and carrots (*Daucus carota* L.). Plastic cylinders plugged with cotton balls were used as water sources. During the experiments, insects were reared in transparent ventilated plastic cylinders (diameter and height of 12 cm) at the temperature of 25 °C and were fed by the same diet.

### 2.2. Nymphal Development

To obtain nymphs, egg masses produced within 24 h by *H. halys* females of the two studied laboratory populations were collected and kept under the pre-set experimental conditions (25 °C; L:D = 16:8) in ventilated transparent plastic cylinders (diameter and height of 12 cm). Every day at 4–6 h after the onset of daily illumination, groups of 25 nymphs of the second instar were randomly selected from a pool of individuals that had molted within 24 h; these groups of 25 nymphs were evenly distributed between the two experimental photoperiods (L:D = 15:9 and 15.5:8.5 at 25 °C) and were reared in the same containers as described above. These near-threshold photoperiods were chosen based on the results of our previous studies performed following a similar experimental protocol [33,39]. Fresh food and water were provided every 2–3 days and the emergence of adults was recorded daily. By this method, the time of development from the beginning of the second instar to the adult stage was determined for 697 individuals (at least 70 insects from each population at each photoperiod). Our previous study [39] showed that the mean duration of pre-adult development did not differ significantly between males and females. Therefore, data for the sexes were pooled.

### 2.3. Reproductive Organs and Fat Body State of Adults

To start this experiment, groups of 3–5 adults of both sexes that had emerged within 2–3 days were reared under the same photo-thermal conditions and on the same diet as during their 2nd–5th nymphal instars (as described above). At 25 days after emergence, all surviving adults were frozen and dissected. This age at dissection is about 1.5 times longer than the mean pre-oviposition period of *H. halys* under the long-day conditions at 25 °C [33,39]. At dissection, the state of reproductive organs and fat body of males and females was recorded on the binary scale used for *H. halys* and other heteropteran species by previous authors [33,38,39,41,50,51,52]. Adults with fully developed reproductive organs (females with mature eggs or vitellogenic oocytes in their ovarioles and males with secretory fluids in their ectodermal sacs of the accessory glands) were considered to be in a reproductive (i.e., nondiapause) state. Otherwise, individuals were considered as diapausing. The fat body state was also evaluated using a binary scale: either fully developed (massive and dense) or poorly developed (loose and depleted). At least 24 adults of each sex from each population per each photoperiod were dissected.

### 2.4. Statistical Analysis

Parametric data (the duration of nymphal development) were analyzed by two-way ANOVA that analyzed the main effects of photoperiod and population origin, as well as their interaction. Nonparametric data (percentages of individuals with fully developed reproductive organs and fat body) were analyzed by two-way probit analysis with photoperiod and population origin as the two factors and by the Spearman correlation analysis. Pairwise comparisons of parametric data were made by the Student’s *t*-test; pairwise comparisons of nonparametric data were made by the χ^2^ test. All above-mentioned calculations were made with SYSTAT 10.2 software (Systat Software Inc., Richmond, VA, USA) [53].

## 3. Results

### 3.1. Nymphal Development

**Figure 3:**
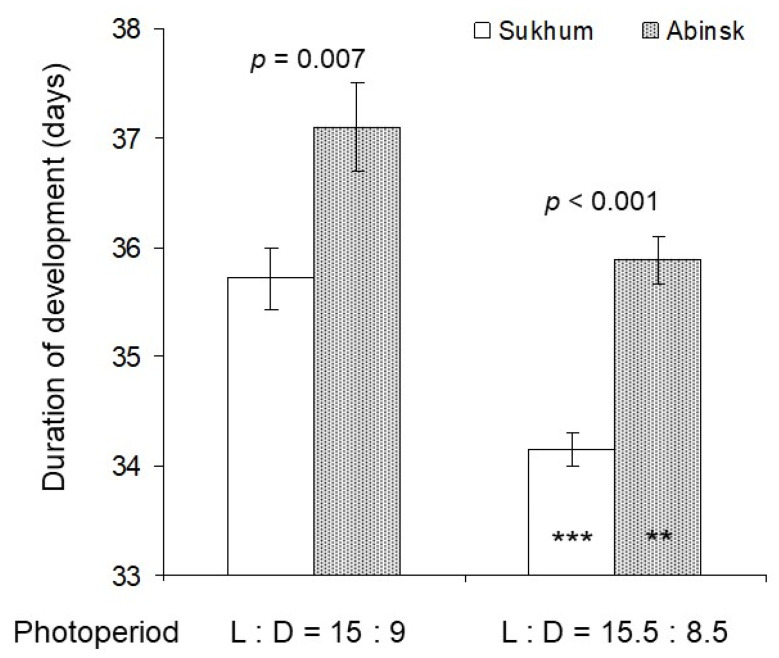
The duration of development from the beginning of the second nymphal instar to the molting to the adult stage in *Halyomorpha halys* from two laboratory populations reared at two photoperiods. Means ± SEM are shown (*n* = 73–265 per bar). Above each pair of bars, significance of the interpopulation difference, i.e., significance of the difference between the data for individuals from different populations reared under the same photoperiod (estimated by the Student’s *t*-test), is given. Asterisks on the two right bars indicate significant photoperiodic response, i.e., significant difference between the data for individuals from the same population reared under two different photoperiods (**—*p* < 0.01, ***—*p* < 0.001 by the Student’s *t*-test).

Two-way ANOVA (*n* = 697) showed that both photoperiod (*F* = 29.2, df = 1, *p* < 0.001) and population origin (*F* = 36.8, df = 1, *p* < 0.001) significantly influenced the time of *H. halys* development from the second instar to the adult stage whereas the interaction of the two factors was not significant (*F* = 0.5, df = 1, *p* = 0.477). In particular, experiments demonstrated that (1) the nymphal development of individuals from the Sukhum population was significantly faster than that of individuals from the Abinsk population and (2) at L:D = 15.5:8.5 nymphs from both populations developed faster than at L:D = 15:9 (Figure 3).

### 3.2. Reproductive Organs and Fat Body Development

**Figure 4:**
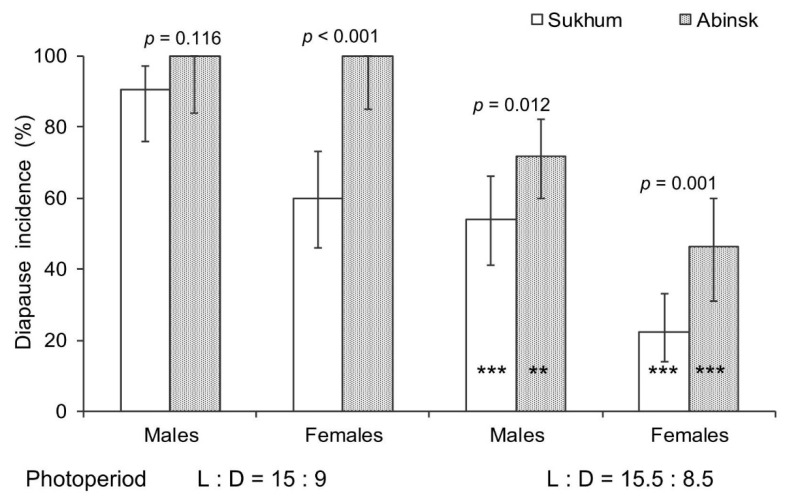
The incidence of diapause in *Halyomorpha halys* males and females from two laboratory populations reared under two photoperiods. Percentage and 95% confidence intervals are presented (*n* = 24–98 per bar). Above each pair of bars, the significance of the interpopulation difference, i.e., the significance of the difference between the data for individuals of the same sex from different populations reared under the same photoperiod (estimated by the χ^2^ test), is given. Asterisks on the four right bars indicate significant photoperiodic response, i.e., significant difference between the data for individuals of the same sex from the same population reared under different photoperiods (**—*p* < 0.01, ***—*p* < 0.001 by the chi-square test).

As seen from the results of the binary probit analysis (Table 1), both photoperiod and population origin have a strong impact on the proportion of *H. halys* males and females with fully developed reproductive organs. The interpopulation difference is clearly seen (Figure 4): under both photoperiods, the proportion of diapausing individuals among males and females from the Abinsk population was significantly higher than among individuals from the Sukhum population. Pronounced photoperiodic response was also evident: the day length of 15.0 h induced diapause in a significantly higher proportion of males and females from the two populations than the day length of 15.5 h.

The degrees of development of reproductive organs and of fat body were negatively correlated (Table 2). Both males and females typically had either poorly developed reproductive organs and fully developed fat body or fully developed reproductive organs and poorly developed fat body, although the other two combinations were also observed. This negative correlation agrees with the results of the probit analysis: the sign of the photoperiodic effect on the fat body development is opposite to that of the effect on the development of reproductive organs (Table 1). Interpopulation difference in the development of the fat body of females is also opposite to that in the development of their ovaries.

## 4. Discussion

This comparative study clearly demonstrated that despite a rather short distance between the two collection sites (ca. 300 km; Figure 1), the two invasive populations of *H. halys* differed in both nymphal development (Figure 3) and diapause incidence (Figure 4) under the two critical photoperiods. The nymphs of the Abinsk population developed more slowly and more frequently entered adult (reproductive) diapause under both photoperiods, although the difference in male diapause incidence between the two populations was not significant under a photoperiod of L:D = 15:9. In addition, bugs from both populations demonstrated similar photoperiodic quantitative and qualitative responses: at the day length of 15.5 h, pre-adult development was faster and the proportion of diapausing adults was lower than those at the day length of 15.0 h. Similar photoperiodic responses have been found in individuals from the earlier studied invasive populations of *H. halys* at Basel, Torino, and Sochi [33].

Our data on the time of *H. halys* pre-adult development are close to those observed in earlier studies of the same species in different parts of the world [35,37,54,55,56,57]. The influence of day length on the rates of growth and development has been demonstrated for several other true bug species [54,58,59,60,61,62,63]. The patterns of the photoperiodic effects on the duration of development varied among insect species. In insects from the temperate climate zone, short-day photoperiods often accelerate development of the pre-diapause stages, thereby increasing the probability of the timely induction of diapause and hence the chances for successful overwintering. However, in some insects, a quite different pattern of the response was also found: the duration of nymphal development decreased at both short and long photoperiods, but the duration was increased at the intermediate (near-threshold) day lengths. It was suggested that the decrease in the rate of development at the near-critical days ensures a longer time for a proper response (either diapause-inducing or diapause-averting) [59,60,64]. Earlier, we demonstrated this pattern of the qualitative photoperiodic response for both native and invasive populations of the brown marmorated stink bug [33], but, in the present study, only two photoperiods were used, which does not allow for similar conclusions related to the pattern of an intermediate response in the Sukhum and Abinsk populations of *H. halys*.

Dissections showed that most males and females have either fully developed fat body and poorly developed reproductive organs (diapausing individuals) or vice versa: poorly developed fat body and fully developed reproductive organs (reproductively active individuals). It is well known that poor development of reproductive organs is the main but certainly not the only component of the reproductive diapause syndrome: as a rule, the induction of diapause is accompanied by the accumulation of fat and other nutrients [1,2,3,4,5,6,7]. However, in some individuals, both ovaries and fat body are relatively fully developed. It is likely that these males and females are in a transitional state: their reproductive diapause is currently either just induced or terminated. Regarding the few bugs with poorly developed ovaries and poorly developed fat body, we believe that most of these individuals either have some inherited abnormalities, were infected or were somehow damaged at the nymphal stage.

As expected, the difference in the tendency to enter diapause agrees with the difference in the local climate: in the temperate zone, the tendency to enter winter diapause is stronger than in the subtropics. Indeed, in both Abinsk and Sukhum, the day lengths of 15.0 and 15.5 h used in our study occur in the middle of August (Figure 2a). Additionally, the mean temperature drops below the lower development threshold of *H. halys* (13.3 °C) starting in early October in Abinsk and early November in Sukhum, respectively (Figure 2b). The monthly mean temperatures exceeded the lower development threshold of *H. halys* by about 10 °C in August and 5 °C in September in Abinsk, while for Sukhum, a difference between such temperatures was approximately 13 °C in August, 9 °C in September, and 4 °C in October. Thus, nymphs of *H. halys* hatched from the eggs laid in the middle of August in Abinsk can count on ca. 45 days of development with the accumulated sum of effective temperatures about 300 degree-days, whereas their peers in Sukhum can count on ca. 75 days of development with the accumulated sum of effective temperatures about 585 degree-days. Our earlier laboratory experiments [39], supported by detailed field observations on the phenology of the Sochi population of *H. halys* [41], indicate that the sum of effective temperatures required for the pre-adult development of this species is about 500–600 degree-days. Hence, nymphs hatched from the eggs laid in the middle of August in Sukhum, such as in Sochi [41], can timely reach the adult stage (and thereby prepare for successful overwintering). However, in Abinsk, the nymphs most probably would not have a chance to successfully reach the adult stage, prepare for overwintering, and survive the winter. Therefore, it is not surprising that at the day length of 15.0 h, almost all females of *H. halys* from the Abinsk population entered winter diapause whereas more than half of the females from Sukhum laid eggs (Figure 4).

Similar and evidently adaptive interpopulation differences in the patterns of diapause-inducing responses have been found by numerous authors in various insect species [3,4,5,7,9,65,66,67]. However, our findings differ from these studies in that the assumed adaptation time was very short. Indeed, most of the earlier studies concerned geographic populations of widely spread but not invasive or rapidly spreading insect species and therefore the adaptation of the compared populations to the local environments lasted for hundreds of years. The brown marmorated stink bug was first recorded in Sukhum in 2015 [32,43] and in Abinsk in 2018 [49]. Most likely the invasion *H. halys* to the region followed the invasion of the pest and its massive spread in Western and Central Europe during the preceding decade [33]. The tested laboratory populations originated from the adults collected in 2020. Thus, it is proposed that the observed difference in the ecophysiological traits between the compared populations resulted from a rapid adaptation that had occurred over a few years.

The extensive ecophysiological literature suggests that, in some other invasive or intentionally introduced insects, significant adaptive interpopulation differences also evolved in a rather limited time. For example, the fall webworm *Hyphantria cunea* Drury (Lepidoptera: Arctiidae) was first discovered in Japan in 1945 and, at that time, only two generations of this invasive lepidopteran pest could be completed per year. However, less than 50 years later in the south-western areas of Japan, most *H. cunea* populations were trivoltine and experiments demonstrated that this change in the seasonal cycle was due to the decrease in the critical photoperiod for the induction of the winter pupal diapause [65]. Similarly, adaptive differences in the photoperiodic induction of diapause were revealed between populations of *Lasiommata megera* (L.) (Lepidoptera: Satyridae), which has naturally spread from Southern to Central Sweden in 2001–2020 [26]. Substantial differences between native and invasive populations have been also found 20 years after the invasion of the multicolored Asian ladybird beetle *Harmonia axyridis* (Pallas) (Coleoptera: Coccinellidae) to Europe. However, in this case, a rather rapid micro-evolutionary adaptation was achieved not by directional changes in the critical day length; instead, the invasive populations decreased their dependence on the photoperiod. Thus, facultative winter adult diapause was induced not by the photoperiodic but rather by the trophic response; namely, by the absence of aphids, which are the natural prey of *H. axyridis* larvae and adults (in the experiment, the green peach aphid *Myzus persicae* (Sulz.) was used) [25]. Adaptive changes in the photoperiodic response of diapause induction in the invasive Asian tiger mosquito *Aedes albopictus* (Skuse) (Diptera: Culicidae) were found 20 years after its invasion to North America. In particular, the changes in the pattern of the critical photoperiod dependence on latitude resulted in a marked decrease in diapause incidence in the southern regions [22]. Moreover, the comparison of four populations of the tamarisk beetle *Diorhabda carinulata* (Desbrochers) (Coleoptera: Chrysomelidae) conducted only 7 years after their introduction from China into North America also demonstrated a latitudinal gradient of the critical day length [19]. Thus, the extremely rapid climatic adaptation of the brown marmorated stink bug revealed in our study is quite unusual but not a unique phenomenon.

Instead of the rapid adaptation to different local climates, it can also be argued that the observed differences in ecophysiological traits resulted from initial genetic dissimilarities caused by separate invasions of donor populations of *H. halys* to the locations. Although further testing is required to rule out this possibility, the crucial role of human activities in the invasion of *H. halys* might be most probable as the primary establishment of the population exclusively in Sochi followed by its subsequent spread. Notably, Sochi is an international Olympic center and a transport hub with large trade and passenger sea ports, an airport, railways, and highways, making it highly likely to be an entry point for the initial invasion and further spread of *H. halys* in the region [32]. In contrast, Abinsk is a small town located on the north-west slope of the Caucasus, and it has limited opportunities for the introduction of new invasive populations of *H. halys* due to its lack of direct connections with other countries. The scenario of invasion of the pest into the Sochi region and subsequent spread is further supported by the recent study which revealed occurrence in Russia of only one cytochrome oxidase I (COI) haplotypes of *H. halys* [68], whereas in some other European countries, populations of this species are much more genetically diverse with occurrence of up to 15 different COI haplotypes per country [69,70,71,72,73,74].

## Figures and Tables

**Table 1:** Influence of photoperiod and population origin on the state of reproductive organs and fat body in *Halyomorpha halys* males (*n* = 259) and females (*n* = 261) as estimated by the binary probit model (data are shown in Table 2).

Factor	Estimated Coefficients (C ± S.E.) and Their Effects (*p)*			
	Influence on the Proportion of Individuals with Fully Developed Reproductive Organs		Influence on the Proportion of Individuals with Fully Developed Fat Body	
	Males	Females	Males	Females
Photoperiod	2.64 ± 0.51, *p* < 0.001	2.47 ± 0.37, *p* < 0.001	−1.04 ± 0.38, *p* = 0.008	−2.62 ± 0.36, *p* < 0.001
Population	−0.53 ± 0.18, *p* = 0.004	−0.89 ± 0.19, *p* < 0.001	0.03 ± 0.17, *p* = 0.853	0.70 ± 0.18, *p* < 0.001

**Table 2:** Development of reproductive organs and fat body in *Halyomorpha halys* males and females in relation to photoperiod and population origin (percentages are given). The Spearman correlation coefficient *ρ* ± S.E., Pearson χ^2^ (df = 1), and sample size (*n*) are calculated for each combination of population origin, photoperiod, and sex.

Fat body	Reproductive Organs			
	Males		Females	
	Poorly Developed	Fully Developed	Poorly Developed	Fully Developed
**Sukhum population, L:D = 15:9**				
Poorly developed (%)	3.8	7.7	2.9	31.4
Fully developed (%)	86.6	1.9	57.1	8.6
Correlation between development of fat body and reproductive organs	*ρ* = −0.699 ± 0.163, χ^2^ = 25.4, *p* < 0.001, *n* = 52		*ρ* = −0.762 ± 0.078, χ^2^ = 40.6, *p* < 0.001, *n* = 70	
**Sukhum population, L:D = 15.5:8.5**				
Poorly developed (%)	12.6	27.6	15.3	67.3
Fully developed (%)	41.4	18.4	7.2	10.2
Correlation between development of fat body and reproductive organs	*ρ* = −0.372 ± 0.100, χ^2^ = 12.0, *p* = 0.001, *n* = 87		*ρ* = −0.206 ± 0.115, χ^2^ = 4.1, *p* = 0.042, *n* = 98	
**Abinsk population, L:D = 15:9**				
Poorly developed (%)	33.3	0.0	15.4	0.0
Fully developed (%)	66.7	0.0	84.6	0.0
Correlation between development of fat body and reproductive organs	– ^a^ *n* = 24		– ^a^ *n* = 26	
**Abinsk population, L:D = 15.5:8.5**				
Poorly developed (%)	12.5	17.7	20.9	37.3
Fully developed (%)	59.4	10.4	25.4	16.4
Correlation between development of fat body and reproductive organs	*ρ* = −0.446 ± 0.100, χ^2^ = 19.1, *p* < 0.001, *n* = 96		*ρ* = −0.245 ± 0.119, χ^2^ = 4.0, *p* = 0.044, *n* = 67	

^a^ Correlation was not calculated because all individuals have poorly developed reproductive organs.

## Data Availability

Data are available upon email request to the corresponding author.
